# Developing a Quantitative Ultrasound Image Feature Analysis Scheme to Assess Tumor Treatment Efficacy Using a Mouse Model

**DOI:** 10.1038/s41598-019-43847-7

**Published:** 2019-05-13

**Authors:** Seyedehnafiseh Mirniaharikandehei, Joshua VanOsdol, Morteza Heidari, Gopichandh Danala, Sri Nandhini Sethuraman, Ashish Ranjan, Bin Zheng

**Affiliations:** 1 School of Electrical and Computer Engineering, University of Oklahoma, 73019 Norman, OK USA; 2 Center for Veterinary Health Science, Oklahoma State University, Stillwater, 74078 OK USA

**Keywords:** Predictive markers, Biomedical engineering

## Abstract

The aim of this study is to investigate the feasibility of identifying and applying quantitative imaging features computed from ultrasound images of athymic nude mice to predict tumor response to treatment at an early stage. A computer-aided detection (CAD) scheme with a graphic user interface was developed to conduct tumor segmentation and image feature analysis. A dataset involving ultrasound images of 23 athymic nude mice bearing C26 mouse adenocarcinomas was assembled. These mice were divided into 7 treatment groups utilizing a combination of thermal and nanoparticle-controlled drug delivery. Longitudinal ultrasound images of mice were taken prior and post-treatment in day 3 and day 6. After tumor segmentation, CAD scheme computed image features and created four feature pools including features computed from (1) prior treatment images only and (2) difference between prior and post-treatment images of day 3 and day 6, respectively. To predict tumor treatment efficacy, data analysis was performed to identify top image features and an optimal feature fusion method, which have a higher correlation to tumor size increase ratio (TSIR) determined at Day 10. Using image features computed from day 3, the highest Pearson Correlation coefficients between the top two features selected from two feature pools versus TSIR were 0.373 and 0.552, respectively. Using an equally weighted fusion method of two features computed from prior and post-treatment images, the correlation coefficient increased to 0.679. Meanwhile, using image features computed from day 6, the highest correlation coefficient was 0.680. Study demonstrated the feasibility of extracting quantitative image features from the ultrasound images taken at an early treatment stage to predict tumor response to therapies.

## Introduction

Before performing clinical trials on cancer patients, mouse models are frequently used as an important step in biomedical research to screen and test new investigative chemotherapy drugs and/or therapeutic methods in order to identify effective drug agents, drug delivery methods, and other treatment technologies for improving the efficacy of cancer treatment^1^. The advantages and necessity of applying mouse models in the initial steps of developing new drugs and/or cancer treatment methods have been extensively investigated and discussed in previous studies^2,3^. As a result, a large number of mouse models bearing different types of simulated carcinoma tumors have been developed and used in cancer research field^4–6^.

In order to non-invasively visualize and characterize tumor response and/or tissue changes during and/or after cancer treatment, medical imaging plays an important role by helping validate certain study hypotheses^7^. Many imaging modalities, such as x-ray imaging including micro-computed tomography (µCT), magnetic resonance imaging (MRI), nuclear and optical imaging, and ultrasound imaging, have been proposed and used for this purpose in the recent years^8–10^. Each imaging modality has its advantages and limitations in predicting or assessing the efficacy of tumor response to the treatment. Compared to other imaging modalities, ultrasound has a number of unique characteristics making it a more attractive tool to predict or assess cancer prognosis to some clinicians. It is a portable, safe (no harmful radiation), easy-to-use, and low-cost imaging modality to assist in monitoring and assessing tumor response and change of tissue characteristics prior and post-treatment^11,12^. However, despite the potential advantages of using ultrasound imaging to assess treatment efficacy, ultrasound often includes higher noise resulting in a relatively low signal-to-noise ratio. Reliably detecting and computing quantitative image features of tumors from ultrasound images is considered more difficult than computing image features from other imaging modalities (i.e., µCT and MRI). As a result, the feasibility of developing or identifying new quantitative imaging markers computed from ultrasound to predict or assess cancer treatment efficacy at an early stage has not been investigated and validated to date.

Thus, based on the concept and scientific premise of Radiomics^13^, the objective of this study is to test the feasibility of identifying and extracting new quantitative image features or markers computed from ultrasound images to predict efficacy of cancer treatment at an early stage. In order to achieve the study objective, we developed an interactive computer-aided detection (CAD) scheme with an easy-to-use graphic user interface (GUI) to process ultrasound images acquired from the colon carcinoma tumor bearing mice and treating with a variety of thermal therapies. From the segmented tumor regions depicted on the ultrasound images, CAD scheme computes a large pool of image features based on tumor morphology, density distribution, and texture related features. Data analysis was then performed to identify top image features and their fusion method to generate new quantitative imaging markers to predict and compare the efficacy of the thermal therapies under tests at an early stage.

## Materials and Methods

In this study, we assembled an experimental dataset that includes 23 athymic nude mice bearing C26 adenocarcinomas. These mice were treated with 7 different thermal based therapies that combine the focused ultrasound-induced mild hyperthermia and chemotherapeutic nanoparticle formulations. Specifically, these 7 treatment methods include (1) high intensity focused ultrasound (HIFU), (2) the chemotherapeutic drug doxorubicin (DOX), (3) DOX and HIFU, (4) low-temperature sensitive liposomes (LTSL), (5) HIFU and LTSL, (6) echogenic low-temperature sensitive liposomes (E-LTSL), and (7) HIFU and E-LTSL. Both LTSL and E-LTSL are different nanoparticle formulations that encapsulate DOX. The details of these treatment methods have been previously reported^14^.

In brief, HIFU is a non-invasive therapeutic technique that uses the focused ultrasound energy to heat a targeted region of tissue in a controlled manner. DOX is a commonly administered, clinically available chemotherapeutic drug, which is often used for treating a wide range of different cancer types. The addition of HIFU hyperthermia increases blood flow to the tumor region and increases drug perfusion in a targeted manner. LTSLs are thermosensitive liposomes that carry a DOX payload to the tumor site; should heating be applied via HIFU (39–42 °C), DOX will be released at tumor site, granting the DOX an additional degree of targeting effect. E-LTSLs have the same lipid composition of the aforementioned LTSL, but also incorporate an ultrasound contrast agent that becomes echogenic (visible on ultrasound) during HIFU heating. They also have the added benefit of promoting improved drug penetration via HIFU/nanobubble interaction.

In the mouse model used in this study, all animal-related procedures were approved and carried out under the regulations and guidelines of the Oklahoma State University Animal Care and Use Committee (ACUP VM-13-24). Specifically, in order to establish a mouse model of colon cancer, C26 cells were grown as a monolayer to 80–90% confluence in RPMI supplemented with 10% v/v fetal bovine serum (FBS) and 1% v/v streptomycin/penicillin. Confluent cells were harvested, washed, and diluted with sterile cold PBS to generate 0.5 × 10^5^ cells/50 μl. Next, 50 μl of cell inoculum was injected per mouse in the thigh region of the mouse hind leg using a 25-gauge needle (BD, Franklin Lakes, NJ, USA). Mice were monitored and tumor growth was measured by serial caliper measurements (General Tools Fraction+^™^, New York, NY, USA). Tumor volumes were calculated using the formula (length × width^2^)/2, where length is the largest dimension and width is the smallest dimension perpendicular to the length. After 3 days, tumors typically grow and reach to a treatment volume of greater than 50 mm^3^ ^14^.

In this study, each mouse was treated twice using the targeted thermal therapy on Day 3 and Day 6 after cell inoculum was injected, respectively. The longitudinal ultrasound images were taken prior and post-treatment in these two days using a Vevo 2100 ultrasound imaging system at a frequency of 21 MHz^14^. During the process, mice were anesthetized and held in custom built mouse holders attached to a 3D positioning stage and the tumors were positioned so that the target was in the center of the focal zone of the ultrasound imaging transducer. In each image acquisition process, multiple ultrasound image frames or a series of imaging video were taken and recorded. Each mouse was monitored for 10 days. At Day 10, the mouse was sacrificed. The tumor was resected and the tumor size was measured.

**Figure 1:**
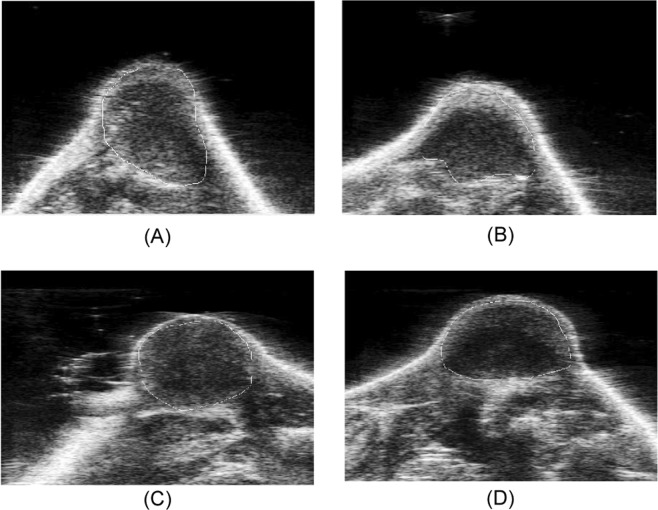
An example of four ultrasound images taken from a mouse in Day 3 (**A**) prior- DOX treatment and (**B**) post-treatment, in Day 6 (**C**) prior-treatment and (**D**) post-treatment, respectively. The tumor boundary contours are marked on each image.

**Figure 2:**
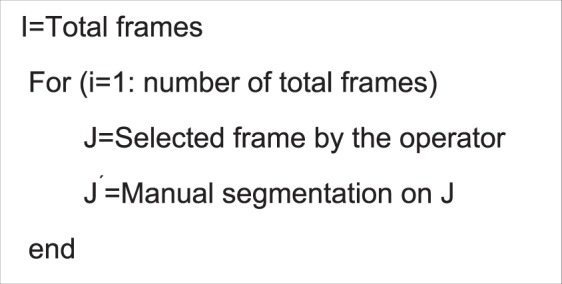
Proposed Algorithm for processing each image.

In this study, we developed an interactive computer-aided detection (CAD) scheme with a graphic user interface (GUI) platform. Following three steps were taken to perform image processing and feature computation. First, after uploading a complete set of ultrasound imaging video series into the GUI, the operator (i.e., a research assistant in the study laboratory) selects one ultrasound image (representing the best frame in the video) in which the tumor area is considered clearly visible. The tumor region is then segmented manually from the ultrasound image. Figure 1 illustrates an example of the tumor regions and their boundary contours segmented from 4 sets of ultrasound images acquired from one mouse prior and post-DOX treatment in Day 3 and Day 6 using the GUI of our CAD scheme, respectively. Specifically, we used the algorithm illustrated in Fig. 2 for processing the images.

**Figure 3:**
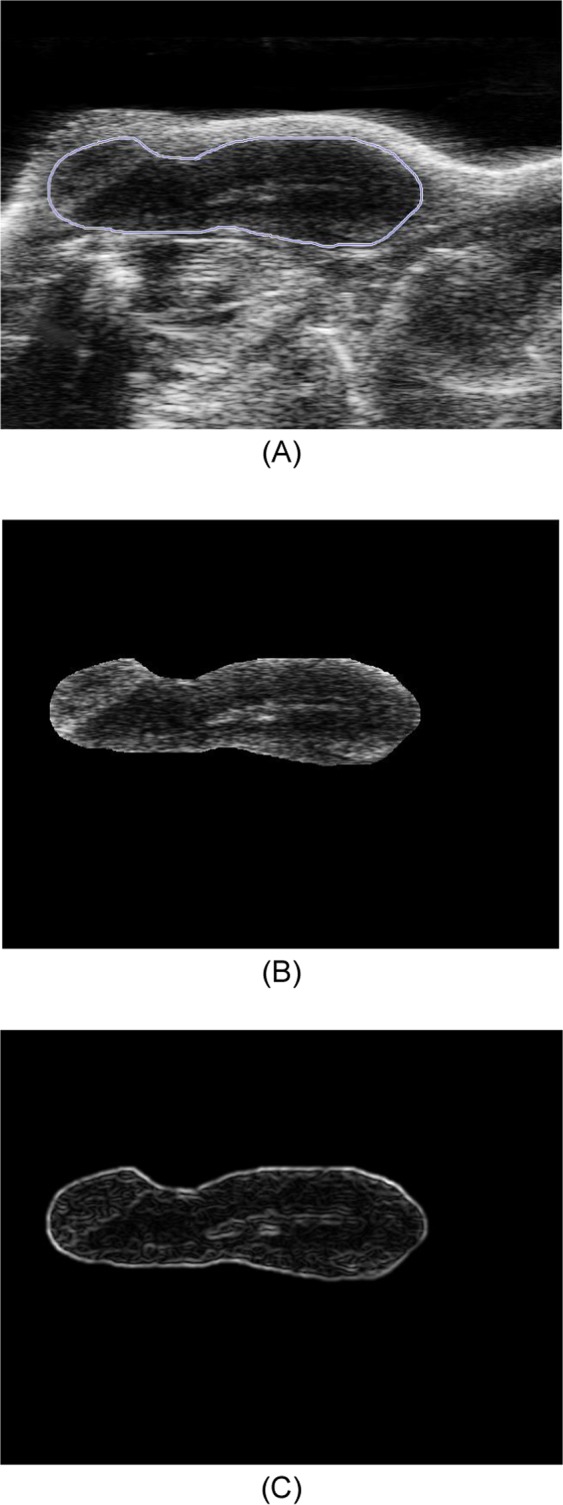
Illustration of applying Gaussian filter to the ultrasound image, which shows (**A**) manually marked tumor boundary contour, (**B**) the segmented tumor region and (**C**) tumor image after applying the Gaussian filter.

After tumor segmentation, the CAD scheme applies a low pass Gaussian filter to the images in order to reduce the inherent noise of the ultrasound images (Fig. 3). CAD then computes image features. A total of 284 image features are computed from each segmented tumor region. The similar tumor-related image features have been computed from other imaging modalities (i.e., CT and MRI) in our previous studies to develop quantitative image markers for predicting tumor response to chemotherapies of treating breast and ovarian cancer^15,16^. These features can be categorized into 4 groups as summarizied in Table 1, which include (1) 9 morphology-based image features; (2) 21 tumor density distribution related image features; (3) 44 grayscale run length (GSRL) based texture related image features^17^, which include (a) Short Run Emphasis (SRE), (b) Long Run Emphasis (LRE), (c) Gray-Level Nonuniformity (GLN), (d) Run Length Nonuniformity (RLN), (e) Run Percentage (RP), (f) Low Gray-Level Run Emphasis (LGRE), (g) High Gray-Level Run Emphasis (HGRE), (h) Short Run Low Gray-Level Emphasis (SRLGE), (i) Short Run High Gray-Level Emphasis (SRHGE), (j) Long Run Low Gray-Level Emphasis (LRLGE), and (k) Long Run High Gray-Level Emphasis (LRHGE) computed in four different directions (0°, 45°, 90°, and 135°), respectively; and (4) 210 image features computed from the wavelet transformation maps.

**Table 1:** List of the computed 284 image features in four feature groups.

Feature Class	Feature Number	Feature Description
Morphology	1–9	Volume, convexity, maximum radius, radius standard deviation (STD), surface area, compactness, maximum three-dimensional diameter, spherical disproportion, and spherical ratio.
Density	10–30	Density, density STD, gradient mean, gradient STD, ISO-intensity, fluctuation mean, fluctuation STD, mean contrast, contrast, skewness, kurtosis, STD ratio of tumor to the boundary, energy, entropy, maximum intensity, mean absolute deviation, median, minimum, range, RMS, and uniformity.
Texture	31–74	11 gray-level run length-based features in four directions (0°, 45°, 90°, and 135°).
Wavelet	75–284	Apply the density and texture features on the four wavelet decompositions.

**Figure 4:**
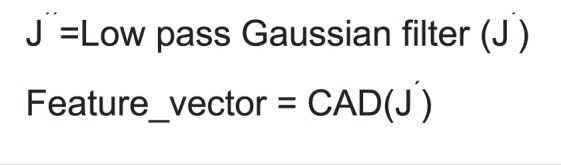
The proposed algorithm for image filtering and feature computation.

Specifically, to compute image features in group 4, CAD applies the wavelet transform on the ultrasound image so that the image is decomposed into four components including I_LL_, I_LH_, I_HL_, and I_HH_, where H and L are labeled as the high- or low-scale decomposition in either the X or Y direction. Intrinsically, in this computation, I_HL_ denotes the component after applying the high-scale and low-scale filter along the X and Y directions, respectively. For each component, the density and texture features measured in the second and third group are recalculated, respectively. Figure 4 shows a two-step algorithm to filter images and compute image features.

After image processing and feature computation, we assembled 4 initial image feature pools, which include the image features computed from (1) prior treatment on Day 3, (2) the difference between prior and post-treatment on Day 3, (3) prior treatment on Day 6, and (4) the difference between prior and post-treatment of Day 6. Afterward, all computed features in each feature pool were normalized to the values between 0 and 1. Hence, for each thermal treatment in Day 3 or Day 6, two initial feature pools were established. The first one includes image features extracted from prior treatment ultrasound images only and the second one includes image feature difference by subtraction between two features computed from the two matched images acquired prior and post-thermal therapy of the same mouse.

**Figure 5:**
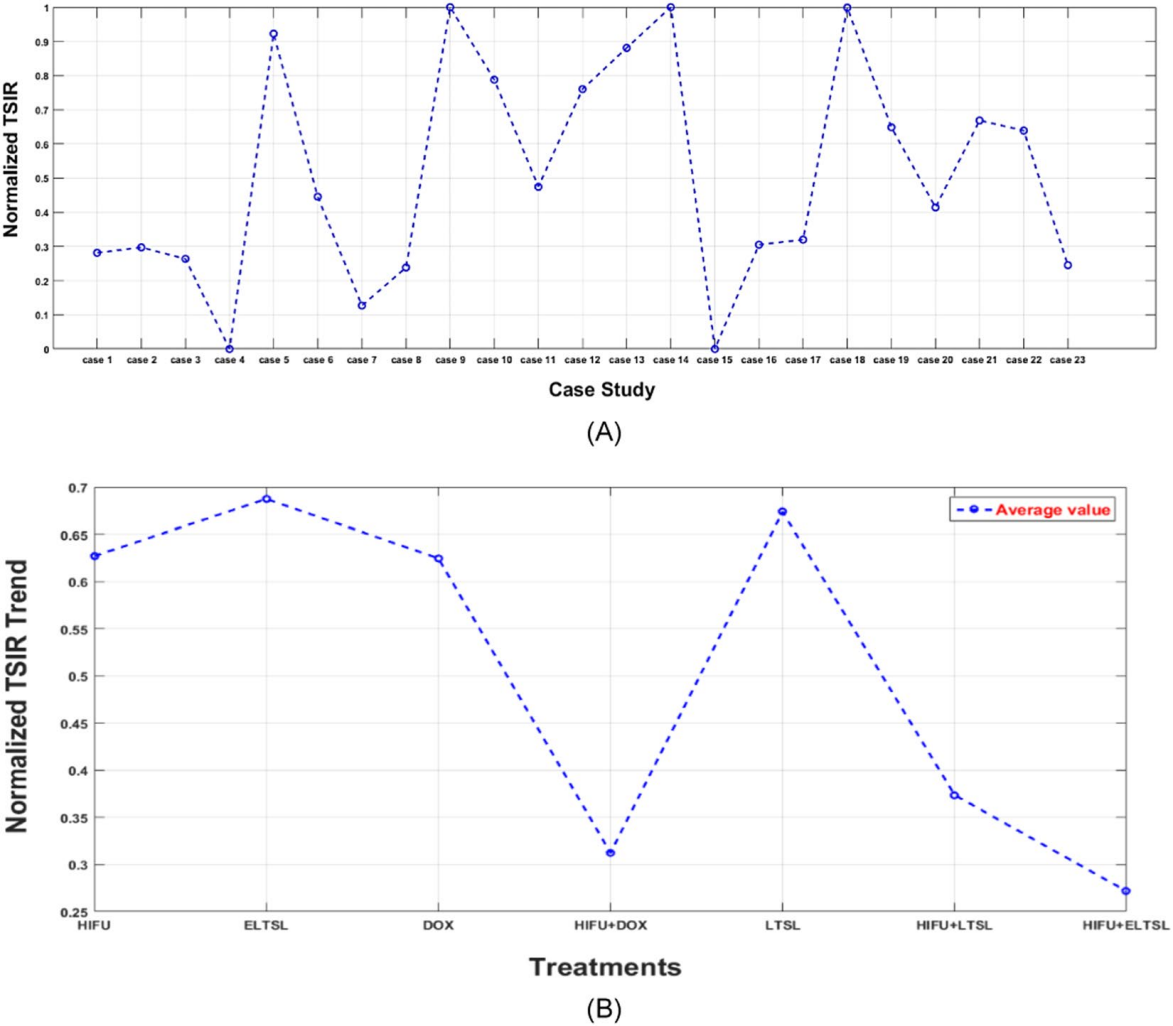
Distribution of the normalized TSIR ratios based on (**A**) each mouse and (**B**) average of each therapy group.

In order to identify and select the potentially effective quantitative image features or markers, we used tumor size change during the period of starting tumor treatment (Day 3) to the end of monitoring (Day 10) as a comparison reference (“ground-truth” of treatment efficacy) in this study. Specifically, the tumor size increment ratio (TSIR) for each mouse during this period is computed with the following formula.

$${\rm{TSIR}}=\frac{x2-x1}{x2}\,\ast \,100$$

where x1 and x2 is the mouse tumor size at day 3 and day 10, respectively. This TSIR based evaluation criterion is similar to Response Evaluation Criteria in Solid Tumors (RECIST) guidelines used in current clinical practice to assess tumor response to therapies among the cancer patients^18^. Then, the computed TSIR were normalized between 0 and 1. Figure 5 shows the normalized TSIR for all 23 mice and is averaged among all therapy groups that were used in this study.

In order to identify the association between the image features and treatment efficacy, we computed the correlation coefficient of each feature with TSIR using the following equation^4^:

$$r=\frac{n({\sum }^{}xy)-({\sum }^{}x)({\sum }^{}y)}{\sqrt{[n{\sum }^{}{x}^{2}-{({\sum }^{}x)}^{2}][n{\sum }^{}{y}^{2}-{({\sum }^{}y)}^{2}]}}$$

where “r” is the Pearson correlation coefficient, “x” and “y” are one selected image feature and the TSIR, respectively. “n” is the case study size, which is 23 in this study. Interpretation of the computed Pearson’s Correlation coefficient to the association between two compared parameters (i.e., one image feature and TSIR in this study) is listed in Table 2 ^19^. Thus, the image features that have higher Pearson’s correlation coefficients with TSIR indicate the higher performance to predict treatment efficacy in this study.

**Table 2:** Pearson Correlation coefficient interpretation^19^.

“r “ Value	Relation
+0.70 or higher (−0.70 or lower)	Very strong positive (negative) relationship
+0.40 to +0.69 (−0.40 or −0.69)	Strong positive (negative) relationship
+0.30 to +0.39 (−0.30 or −0.39)	Moderate positive (negative) relationship
+0.20 to +0.29 (−0.20 or−0.29)	Weak positive (negative) relationship
+0.01 to +0.19 (−0.01 or −0.19)	No or negligible relationship
0	No relationship [zero correlation]

By computing Pearson’s correlation coefficients of 284 image features stored in each of the 4 initial feature pools representing the ultrasound imaging tests performed on Day 3 and Day 6, we first selected the top five image features that have a higher correlation with TSIR in each feature pool. Next, we calculated the correlation coefficient of these five features with each other. Then, in order to reduce redundancy, we selected two features among these 5 top features, which have the lowest correlation coefficient, to generate a new fusion marker using an equally weighting method, ${F}_{new}=({F}_{1}+{F}_{2})/2$. Similar fusion method has been used in our previous studies (i.e.^20^).

**Figure 6:**
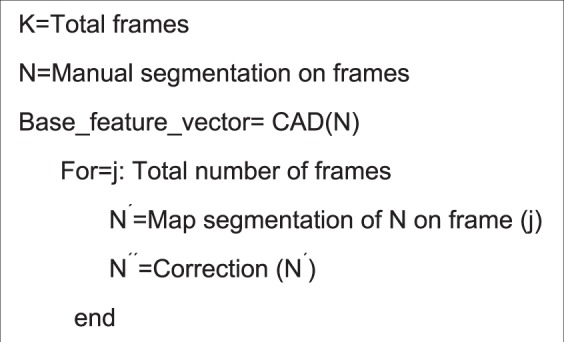
The proposed algorithm for examining reproducibility or consistency between the image features computed from the base frame and other frames.

We also recognize that unlike other imaging modalities (i.e., CT or MRI), an ultrasound imaging test usually acquires multiple image frames. In this study, one ultrasound imaging test or scan typically includes up to 200 image frames. In order to test scientific rigor or reproducibility of the quantitative image features computed from different ultrasound image frames, we defined one frame originally selected by the operator of the GUI of our CAD scheme as the base frame of a set of ultrasound images acquired in one test. We also processed and computed the same image features of the segmented tumor region from all other ultrasound image frames (i.e., the remaining 199 frames) in this set. Then, we computed the mean correlation coefficient and the standard deviation of the features computed from the base frame and all other 199 frames. Figure 6 shows an algorithm to examine the reproducibility of the features computed from the base frame as compared to the features computed from all other frames.

## Results

Table 3 shows two sets of 5 optimal image features with the highest Pearson correlation coefficients with the treatment outcome (TSIR) and the corresponding p-values. These features are selected from the two initial image feature pools that record the image features computed from the ultrasound images acquired prior treatment on Day 3 and Day 6, respectively. It shows that using image features computed from prior treatment ultrasound images acquired on Day 3 yielded a moderate correlation and there are no significant differences between the top 5 features (p > 0.05), while using the image features computed from prior treatment ultrasound images acquired on Day 6 increase the correlation level to a strong positive correlation and the number one feature yielded significantly higher correlation as compared to other 4 top features (p < 0.01).

**Table 3:** List of two sets of the selected 5 top image features from 2 image features of prior treatment on Day 3 and Day 6.

Day 3		P value comparing to Range	Day 6		P value comparing to GLN_HL_
Feature	Correlation coefficient with TSIR		Feature	Correlation coefficient with TSIR	
Range	**0**.**375**		GLN_HL_	**0**.**680**	
Entropy_HH_	0.361	0.468	RP_HL_	0.643	<0.01
RP_LL_	0.359	0.415	GLN_HL_ 90°	0.605	<0.01
Entropy_LL_	0.355	0.478	RLN_LL_	0.598	<0.01
GLN_HL_ 90°	0.344	0.377	Entropy_HL_	0.597	<0.01

The top 5 performed image features selected on Day 3 and Day 6 are different as shown in Table 3, which indicates that treatments have an impact on the change of tumor morphological and texture characteristics. In addition, Table 4 shows and compares 5 sets of correlation coefficients of the same image features computed from prior treatment ultrasound images acquired on both Day 3 and Day 6. The results show that image features contain increased discriminatory power or higher correlation coefficients as they approach the endpoint of Day 10 (i.e., Day 6 vs. Day 3) to predict treatment efficacy or outcome.

**Table 4:** Comparison of the correlation coefficients of the same image features computed from prior treatment ultrasound images acquired on Day 3 and Day 6.

	GLN_HL_ 90°	Tumor Volume	RLN_HL_	RP_HH_ 90°	GLN_LL_
Day3	0.344	0.341	0.329	0.326	0.318
Day6	0.605	0.525	0.586	0.546	0.551

Table 5 shows the correlation coefficients between TSIR and 5 top image features selected from the feature pool that contains image feature difference computed between prior and post-treatment ultrasound images acquired on Day 3. It shows that using the image features that represent the difference of the tumor response or characteristic change prior and post-therapies yield higher correlation with TSIR or higher prediction power. In addition, by selecting two of the 5 top features (as listed in Table 5), which have the smallest correlation coefficients among the 5 top features, we applied an equally weighted fusion method to generate a new image marker. The correlation coefficient of this new fusion based image marker and TSIR significantly increased to 0.679 as shown in Table 5.

**Table 5:** List of the 5 selected image features computed from the difference of prior and post-treatment ultrasound images acquired on Day 3 with the high correlation with TSIR.

No.	Features	Correlation coefficient with TSIR	P value comparing to F1
F1	GLN_HL_	**0**.**552**	
F2	LGRE 0°	0.495	0.128
F3	Range_HL_	0.388	0.011
F4	LGRE_LL_	0.387	0.530
F5	Gradient STD_LH_	0.373	0.809
	Fusion Average (F1 & F3)	**0**.**679**	**<0**.**01**

Table 6 shows the 5 selected image features computed from the differences between the prior and post-treatment ultrasound images acquired on Day 6 with the highest correlation coefficients to TSIR. However, when comparing the correlation coefficients of the top 5 features computed from prior treatment ultrasound images acquired on Day 6 (Table 3), correlation coefficients decrease, which indicates that adding the image features computed from post-treatment ultrasound images on Day 6 does not help increase power to predict treatment outcome (TSIR determined on Day 10).

**Table 6:** The top image features computed from the difference of prior and post-treatment ultrasound images acquired on Day 6 with the high correlation with TSIR.

Feature	Correlation coefficient with TSIR	P value comparing to RLN_HH_ 45°
RLN_HH_ 45°	**0**.**428**	
RLN_HH_ 135°	0.420	<0.01
RP_HH_ 90°	0.358	<0.01
RP_HH_	0.357	<0.01
LGRE_LL_	0.306	0.251

**Figure 7:**
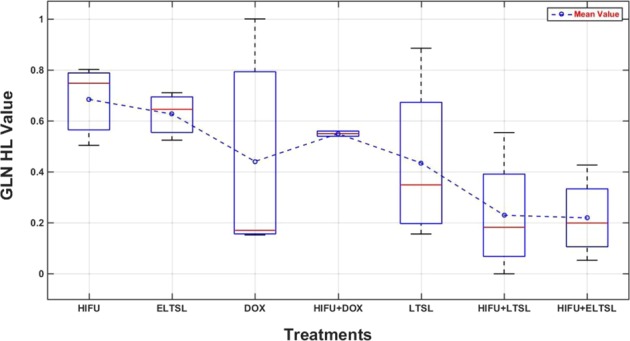
The GLN_HL_ values computed from all mice under different treatments, which are sorted from low to high performance (right to left), respectively.

Subsequently, we separately analyzed and sorted feature distribution in 7 groups of different thermal therapy methods. Each of the 7 treatment groups involves 2 to 4 mice. For example, Fig. 7 shows the distribution (or boxplot) of one of the top image features (the GLN_HL_ values) computed from the difference of prior and post-treatment ultrasound images acquired on Day 3 across the treatment groups. The result shows a trend indicating that the image feature values vary when using different thermal therapy methods. Finally, Table 7 shows examples of the mean correlation coefficient, standard deviation and 95% confidence interval of the base frame and the other 199 frames for the top five image features computed from the difference of prior and post-treatment ultrasound images acquired on Day 3. These image features were computed from a mouse within the HIFU+ELTSL treatment group. The results show the image features computed from different frames of ultrasound images acquired in one test are highly correlated or invariant.

**Table 7:** An example of the base frame and other frames relationships.

	Mean Correlation Coefficient	95% Confidence Interval	Standard deviation
GLN_HL_	0.978	[0.951, 1.00]	0.125
LGRE 0°	0.9668	[0.019, 0.917]	0.224
Range_HL_	0.9491	[−0.058, 0.608]	0.167
LGRE_LL_	0.9719	[0.100, 0.978]	0.219
Gradient STD_LH_	0.9513	[0.089, 0.905]	0.204

## Discussion

In cancer research, many previous studies have reported to develop and apply either molecular biomarkers (i.e.,^21–23^) or quantitative image markers (i.e.^15,16,24–26^) to predict tumor response to chemotherapies and/or other therapeutic methods at an early stage. In this study, we investigated and demonstrated the feasibility of identifying new quantitative image markers computed from ultrasound images. This study has a number of unique characteristics and potential impacts. First, in the previous studies, quantitative image markers were computed based on Radiomics concept that uses CT and/or MRI images to predict cancer prognosis or tumor response to treatment^13^. In this study, we applied the Radiomics approach to ultrasound images to identify new quantitative image markers to predict the efficacy of cancer treatment. Due to the advantages of ultrasound imaging as a diagnostic modality, developing highly performed and robust image markers could be a cost-effective approach in future research and clinical service. Second, thermal based therapies have been emerging as a promising cancer treatment method. However, accurate prediction aimed at determining efficacy or treatment outcomes of different therapeutic approaches remains an unsolved challenge. This is the first study that presents a computer-aided approach to develop new quantitative imaging markers that can predict the efficacy of thermal therapies using a mouse model. Third, we computed and compared image features of the longitudinal images acquired not only from prior and post-treatment ultrasound images but also from two-time points (Day 3 and Day 6 after initial tumor cell embedment). As a result, we are able to conduct a more comprehensive data analysis and identify an optimal approach to extract and compute image markers in order to more accurately predict treatment efficacy or outcome at an early stage.

From the experiments and data analysis results, we can make the following new observations. First, this study shows that it is possible to identify quantitative ultrasound image feature based markers at an early stage (i.e., Day 3 in this study) to predict thermal therapy efficacy. However, in the early stage, using the image marker computed from both prior and post-treatment ultrasound images can yield substantially higher prediction accuracy as compared to using the prior treatment images only (i.e., correlation coefficients of 0.375 vs. 0.679 as shown in Tables 3 and 5). This observation is consistent with our previous study of developing quantitative image markers computed from prior and post-chemotherapy CT images to predict the response of ovarian cancer patients to chemotherapy in the clinical trials^16^.

Second, we observed that using quantitative image markers computed from the prior treatment ultrasound images acquired on Day 6 yielded substantially higher prediction power than the image markers computed from the prior treatment ultrasound images acquired on Day 3. The correlation coefficients increase from the moderate level to the strong positive level for both different top 5 features as shown in Table 3 and the same highly performed features as shown in Table 4. Thus, the trend is consistent, which indicates that the images acquired at the later stage (i.e., Day 6 in this study) contain higher discriminative information or predictive power to evaluate treatment outcome.

Third, we also observed a different phenomenon when using the image features computed from the difference between prior and post-treatment ultrasound images acquired on Day 3 and Day 6. Unlike the image markers computed on Day 3, adding post-treatment ultrasound images acquired on Day 6 does not further increase prediction power or have lower correlation coefficients as shown in Table 6. It reveals that in the earlier day (Day 3), the tumors have higher positive responses to the thermal therapies than those in the later day (Day 6), which may indicate that at the later stage, tumors are more resistant to the treatment. Thus, the observation may clearly show that early treatment (i.e., on Day 3) is more important and effective than later treatment (i.e., on Day 6 in this study), which is consistent with the established scientific evidence in cancer treatment research.

Fourth, the computed image feature values also vary on the ultrasound images acquired from the mice under different treatment methods. In this study, 23 mice were treated with 7 different thermal therapies. Based on the final tumor size measurement results or TSIR on Day 10, the effectiveness of these 7 thermal methods has a monotonically decreased trend from thermal therapy method 1 (HIFU) to method 7 (HIFU + E-LTSL). The computed image features also show a similar trend as shown in Fig. 7, which indicates the high correlation between the image features and thermal therapy methods. Thus, using quantitative image markers also has the potential to help identify optimal therapy methods.

Last, although computer-aided detection schemes of medical images can be quite sensitive to change of image noise^27^, we observed that image features computed from all image frames (i.e., 200 in this study) acquired at one ultrasound imaging test of a mouse were highly correlated or invariant to the small change of inherent image noise. As an example shown in Table 7, the mean correlation coefficients of the top five image features computed from the base image frame and other 199 image frames in one ultrasound imaging scan of a mouse ranged from 0.949 to 0.978. The results revealed that as long as using a well-established or controlled imaging protocol, it is feasible to robustly compute quantitative image features from the ultrasound images for predicting the efficacy of tumor response to therapies.

Despite the promising data analysis results and observations, this is a preliminary study with limitations. For example, in this study, we only used a small dataset including 23 athymic nude mice, which were divided into 7 groups treated with 7 different thermal therapies. The ultrasound images were acquired by one group of researchers in one research laboratory. Thus, in order to validate the study results and enhance the feasibility of developing robust image feature based markers to predict cancer treatment efficacy, more studies are needed by using larger and diverse datasets from both prior clinical (using mouse models) and clinical (using real patient images) researches in the future.

## Conclusion

Ultrasound is a safe, easy-to-use, and low-cost medical imaging modality. In this study, we investigated the feasibility of identifying and applying quantitative image feature based markers computed from ultrasound images using a C26 adenocarcinoma bearing mouse model to assess tumor response to thermal therapies. The study results demonstrated that ultrasound images acquired prior and post-therapy at an early stage (i.e., Day 3 of this study) contained useful and highly discriminative information that can possibly predict tumor response to therapies at an early stage. Although ultrasound images may have a higher inherent noise level than other medical imaging modalities (i.e., CT or MRI), this study also indicated the possibility of computing highly robust image features for the task of developing robust image feature based markers. Thus, based on the preliminary results of this study, future studies using larger and more diverse image datasets are needed to further validate the performance and potential utility of this methodology and similar approaches.
